# Long distance transport of irradiated male *Glossina palpalis gambiensis* pupae and its impact on sterile male yield

**DOI:** 10.1186/s13071-015-0869-3

**Published:** 2015-05-01

**Authors:** Soumaïla Pagabeleguem, Momar Talla Seck, Baba Sall, Marc JB Vreysen, Geoffrey Gimonneau, Assane Gueye Fall, Mireille Bassene, Issa Sidibé, Jean-Baptiste Rayaissé, Adrien MG Belem, Jérémy Bouyer

**Affiliations:** Pan-African Tsetse and Trypanosomosis Eradication Campaign, Bobo-Dioulasso, 01 BP 1087 Burkina Faso; Centre de Coopération Internationale en Recherche Agronomique pour le Développement, Unité Mixte de Recherche Contrôle des Maladies Animales Exotiques et Emergentes, Campus International de Baillarguet, Montpellier, 34398 France; Institut National de la Recherche Agronomique (INRA), Unité Mixte de Recherche 1309 ‘Contrôle des Maladies Animales Exotiques et Emergentes’, Montpellier, 34398 France; Institut Sénégalais de Recherches Agricoles, Laboratoire National d’Elevage et de Recherches Vétérinaires, Service de Parasitologie, Dakar - Hann, BP 2057 Sénégal; Direction des Services Vétérinaires, 37, avenue Pasteur, Dakar, BP 67 Sénégal; Insect Pest Control Laboratory, Joint FAO/IAEA Programme of Nuclear Techniques in Food and Agriculture, Wagramer-strasse 5, PO Box 100, Vienna, A-1400 Austria; Centre International de Recherche-développement sur l’Élevage en Zone Subhumide, Bobo-Dioulasso, 01 BP 454 Burkina Faso; Université Polytechnique de Bobo-Dioulasso, Houet, BP 1091 Burkina Faso; Centre de Coopération Internationale en Recherche Agronomique pour le Développement (CIRAD), Unité Mixte de Recherche ‘Interactions hôtes-vecteurs-parasites-environnement dans les maladies tropicales négligées dues aux trypanosomatides’, Montpellier, 34398 France

**Keywords:** Area-wide integrated pest management, Sterile insect technique, Pupae development, Low temperatures, Pupae transport, Mass-rearing, Diptera, Glossinidae

## Abstract

**Background:**

The application of the sterile insect technique (SIT) requires mass-production of sterile males of good biological quality. The size of the project area will in most cases determine whether it is more cost effective to produce the sterile flies locally (and invest in a mass-rearing facility) or import the sterile flies from a mass-rearing facility that is located in another country. This study aimed at assessing the effect of long distance transport of sterile male *Glossina palpalis gambiensis* pupae on adult male fly yield.

**Methods:**

The male pupae were produced at the Centre International de Recherche-Développement sur l’Elevage en zone Subhumide (CIRDES), Bobo-Dioulasso, Burkina Faso, and shipped with a commercial courier service in insulated transport boxes at a temperature of ±10°C to Senegal (±36 h of transport). Upon arrival in the insectary in Dakar, the pupae were transferred to an emergence room and the flies monitored for 3–6 days.

**Results:**

The results showed that the used system of isothermal boxes that contained phase change material packs (S8) managed to keep the temperature at around 10°C which prevented male fly emergence during transport. The emergence rate was significantly higher for pupae from batch 2 (chilled at 4°C for one day in the source insectary before transport) than those from batch 1 (chilled at 4°C for two days in the source insectary before transport) i.e. an average (±sd) of 76.1 ± 13.2% and 72.2 ± 14.3%, respectively with a small proportion emerging during transport (0.7 ± 1.7% and 0.9 ± 2.9%, respectively). Among the emerged flies, the percentage with deformed (not fully expanded) wings was significantly higher for flies from batch 1 (12.0 ± 6.3%) than from batch 2 (10.7 ± 7.5%). The amount of sterile males available for release as a proportion of the total pupae shipped was 65.8 ± 13.3% and 61.7 ± 14.7% for batch 1 and 2 pupae, respectively.

**Conclusions:**

The results also showed that the temperature inside the parcel must be controlled around 10°C with a maximal deviation of 3°C to maximize the male yield.

**Electronic supplementary material:**

The online version of this article (doi:10.1186/s13071-015-0869-3) contains supplementary material, which is available to authorized users.

## Background

The western region of Senegal called Niayes is characterized by specific climatic and ecological conditions with good potential for keeping exotic cattle for milk and meat-production. Most of the intensive milk production systems of suburban Senegal that keep mainly exotic cattle breeds such as Montbeliard, Jersiaise, Holstein and Girare found in this area [1,2]. This area is also infested with the tsetse fly *Glossina palpalis gambiensis* Vanderplank (Diptera: Glossinidae) which is the main vector of *Trypanosoma vivax* and *T. congolense* [3], parasites that cause the debilitating disease African Animal Trypanosomosis (AAT) in livestock [4]. In Senegal, the livestock sector is far from satisfying national milk and meat needs and depends on imported products that represent double the national production to meet the demand [2]. The Government of Senegal is making efforts to maximize the potential of this region by stimulating programs that aim at improving animal productivity as to meet the growing internal demand in animal products, improve food and nutrition security and reduce the cost of milk imports due to the presence of trypanosomosis [5-7].

In 2000, the African Heads of State and Government decided to increase efforts to address the tsetse and trypanosomosis problem on the African continent and created the Pan-African Tsetse and Trypanosomosis Eradication Campaign (PATTEC) [8]. In this context, the Government of Senegal initiated in 2005 a program called “Projet d’éradication des mouches tsé-tsé dans les Niayes” [3]. This initiative has been technically and financially supported by the Government of Senegal, the Food and Agriculture Organization of the United Nations (FAO), the International Atomic Energy Agency (IAEA), the Centre de Coopération Internationale en Recherche Agronomique pour le Développement (CIRAD), and the United States State Department under the Peaceful Uses Initiative (PUI). Results from the baseline data collection and the feasibility studies in the Niayes have indicated that *G. p. gambiensis* was the only tsetse species present [5] and that the various populations were genetically isolated from the nearest population in Missira located >200 km to the south-east [9]. In addition, studies carried out in walk-in field cages showed that a Burkina Faso strain of *G. p. gambiensis* was sexually compatible with the populations inhabiting the Niayes [10]. The results of these studies prompted the project stakeholders in 2011 to develop and implement a strategy of eradication following area-wide integrated pest management (AW-IPM) principles whereby several tsetse control tactics would be combined with the sterile insect technique (SIT) [5,11].

Partnerships have been developed with international organizations such as the FAO, the IAEA and the Centre International de Recherche-Développement Sur l’Elevage en Zone Subhumide (CIRDES) and national institute such as the CIRAD. In this context, an agreement was made with the CIRDES in Bobo-Dioulasso, Burkina Faso to mass-produce the Burkina Faso (BKF) strain of *G. p. gambiensis* to supply the sterile males needed for the SIT component of the AW-IPM program in Senegal. This required the long distance transport of male pupae from Bobo-Dioulasso to Dakar via Ouagadougou (2100 km) which could affect the competitiveness of the male flies and the success of the eradication program.

The aim of this work was to assess the effectiveness of the developed pupal transport methods from the source insectary in Bobo-Dioulasso to Dakar and to assess the effect of transport on the number of sterile males available for the SIT component of the programme.

## Methods

### Insectary

The study was carried out in an insectary at the Institut Sénégalais de Recherche Agricoles, Laboratoire National d’Elevage et de Recherches Vétérinaires, Service Bio-Ecologies et de Pathologies Parasitaires (ISRA/LNERV/BEPP) in Dakar. The insectary was equipped to receive and incubate the pupae, to monitor their emergence, and to assess total available sterile male flies and their quality. In the insectary, the flies were maintained at 24-25°C, 75-80% RH, and a photoperiod of 12:12 h (L:D).

### Biological material

Pupae of the *G. p. gambiensis* BKF strain were used in this study. This tsetse colony has been maintained at the CIRDES insectary for more than 40 years and feeding is done using in vitro silicon membrane system using irradiated cow blood that is collected from the local abattoir [12]. The colony was established in 1972 at Maison-Alfort (France) with pupae collected from Guinguette (Bobo-Dioulasso) and in 1975 the colony was transferred to the Centre de Recherche sur la Trypanosomiase Animale (CRTA) (renamed later CIRDES). In 1981 the colony was supplemented with wild material from the “Marreaux hippopotames” [10]. Based upon research carried out at the Insect Pest Control Laboratory (IPCL) [13], a protocol was developed that enabled the shipment of sterile male pupae while retaining the majority of the female flies for colony maintenance. Female tsetse flies emerge before the males [14] and as soon as most of the females had emerged (indicated by the first emergences of males) the remaining pupae were chilled to 4°C to prevent male fly emergence. Ninety percent of pupae were 29 days (29 ± 1) of age when chilling started.

### Packaging and transport of pupae

At the CIRDES insectary, pupae were collected weekly on Wednesday (batch 1) and Thursday (batch 2) from January 2011 to July 2013, and on Sunday (batch 1) and Monday (batch 2) from August 2013 to January 2014. Pupae were immediately chilled (4°C) after collection and irradiated under chilled conditions the following day with 110 Gy in a ^137^Ce source for 24 min 30 seconds. Pupae were transported on Friday (January 2011 to July 2013) and on Tuesday (August 2013 to January 2014) with a courier service (DHL®) using public bus transport from Bobo-Dioulasso to Ouagadougou and commercial aircraft between Ouagadougou and Dakar.

The irradiated pupae were placed in petri dishes at 8-10°C and packed in insulated boxes containing phase change material packs (S8) (PCM Phase Change Material Products limited, Cambridgeshire, United Kingdom) to maintain the temperature around 10°C (see additional file for a detailed description of the packaging protocol). The average transport and chilling time for pupae from batch 1 and 2 was 84 ± 9 and 60 ± 12 hours, respectively i.e. 48 ± 7 and 24 ± 10 hours at 4°C in the source insectary and ±36 hours at ±10°C for transport to Dakar. The box size and the number of S8 packs used were adjusted to the number of pupae shipped. As an example six S8 packs were used for a shipment of 3000 pupae in a box with an internal size of 15x17x19cm. The transport box was accompanied by a document indicating the number and age of the shipped pupae, time of chilling, irradiation dates, irradiation duration and dose. The data obtained from the sheet was used for monitoring the quality of the pupae on arrival at the ISRA insectary.

### Emergence and monitoring of flies at the insectary

The study was implemented from January 2011 to January 2014. At the insectary in Dakar, the pupae of each shipment were placed in Petri dishes and covered with ~1 cm of autoclaved sand mixed with a fluorescent dye (DayGlo®) (0.5 g dye / 200 g of sand) to mimic natural emergence conditions and to allow discrimination with wild flies in the monitoring traps (as these sterile male flies were also used for sterile male release trials and operational releases). Emerged flies of each shipment were chilled between 2 and 4°C every 24 hours and sorted by sex to remove the females that were accidentally included in the shipments. Flies with deformed wings were discarded and “normal” males were kept in standard fly holding cages (20 cm diameter and 7 cm height) at a density of 120 males per cage and offered a blood meal every morning for 3–6 days using an in vitro silicon membrane feeding system [15]. For each liter of blood used for feeding, 10 mg of the trypanocidal drug isometamidium [16,17] was added to prevent the cyclical development of trypanosomes in the released sterile males.

Records were kept of daily mortality of the male flies and of the numbers of flies with deformed wings that had emerged during transport and at the insectary at ISRA. The emergence rate of pupae was calculated as the ratio of the number of flies emerged to the number of pupae received. The percentage of females was calculated in order to have the exact number of male pupae sent. The yield of sterile males of each shipment usable for the releases (i.e. available sterile males) was estimated as the ratio of the usable males to the number of pupae received.

### Temperature and relative humidity records during transport

**Figure 1:**
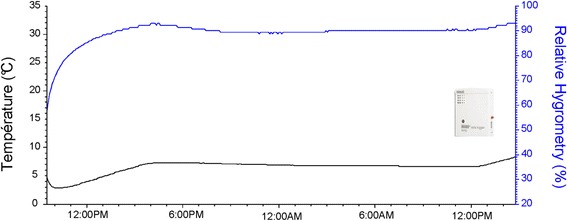
Temperature and relative humidity recorded inside the insulated transport box with a data logger during a pupal shipment from Bobo-Dioulasso to Dakar (10-11/02/2012).

Temperature and relative humidity inside the insulated box were recorded with a Hobo® data logger for each shipment. It was added to the shipping box during the packaging at the CIRDES. The Hobo® was programmed to record data every 5 minutes. After arrival at the ISRA insectary, temperature and relative humidity data recorded during transport were downloaded from the Hobo® with the HOBOware Software (see example Figure 1).

### Data analysis

The adult emergence rate, sterile male mortality rate in the insectary before release, percentage of flies that had emerged with deformed wings, percentage of females and percentage of available sterile males for release were analyzed using binomial mixed effects models. The shipment date was used as a random effect, whereas the batches (batch 1 chilled (4°C) one day longer than batch 2) and the climatic variables (temperature and relative humidity) and their interaction were used as fixed effects. The best model was selected on the basis of the lowest corrected Akaike information criterion (AICc), and the significance of fixed effects was tested using the likelihood test ratio [18,19]. The R (version 2.15.0) Software was used for data analysis [20].

### Ethical statement

The study was conducted in the framework of the tsetse control program in Senegal, led by the Direction of Veterinary Services, Ministry of livestock. This project received official approval from the Ministry of Environment of Senegal, under the permit N°0874/MEPN/DE/DEIE/mbf.

## Results

### Temperature and relative humidity during transport of pupae

**Figure 2:**
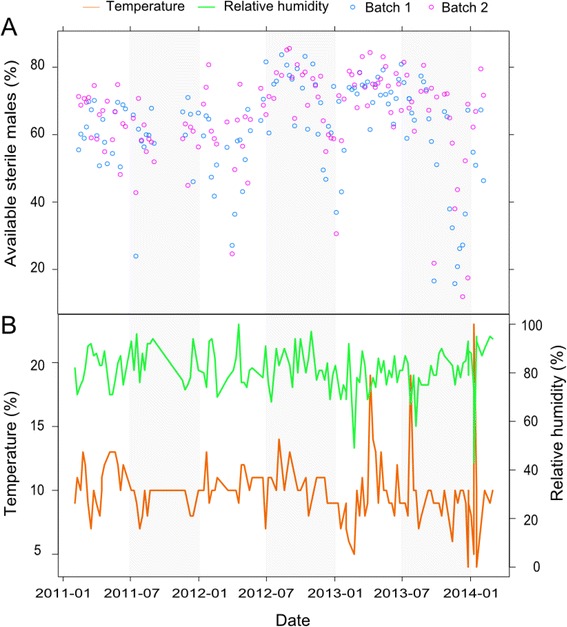
**(A)** The number of sterile males available for release, **(B)** the average temperature (orange line) and relative humidity (green line) during the transport from Bobo-Dioulasso to Dakar.

The average temperature (±sd) inside the insulated transport box of shipments from CIRDES over three years was 10.1 ± 2.3°C (Figure 2) but peaks up to 20°C were recorded. The relative humidity of 92% shipments ranged between 70 and 95% (Figure 2), averaging (±sd) 81.4 ± 8.7%.

### Emergence during transport

**Figure 3:**
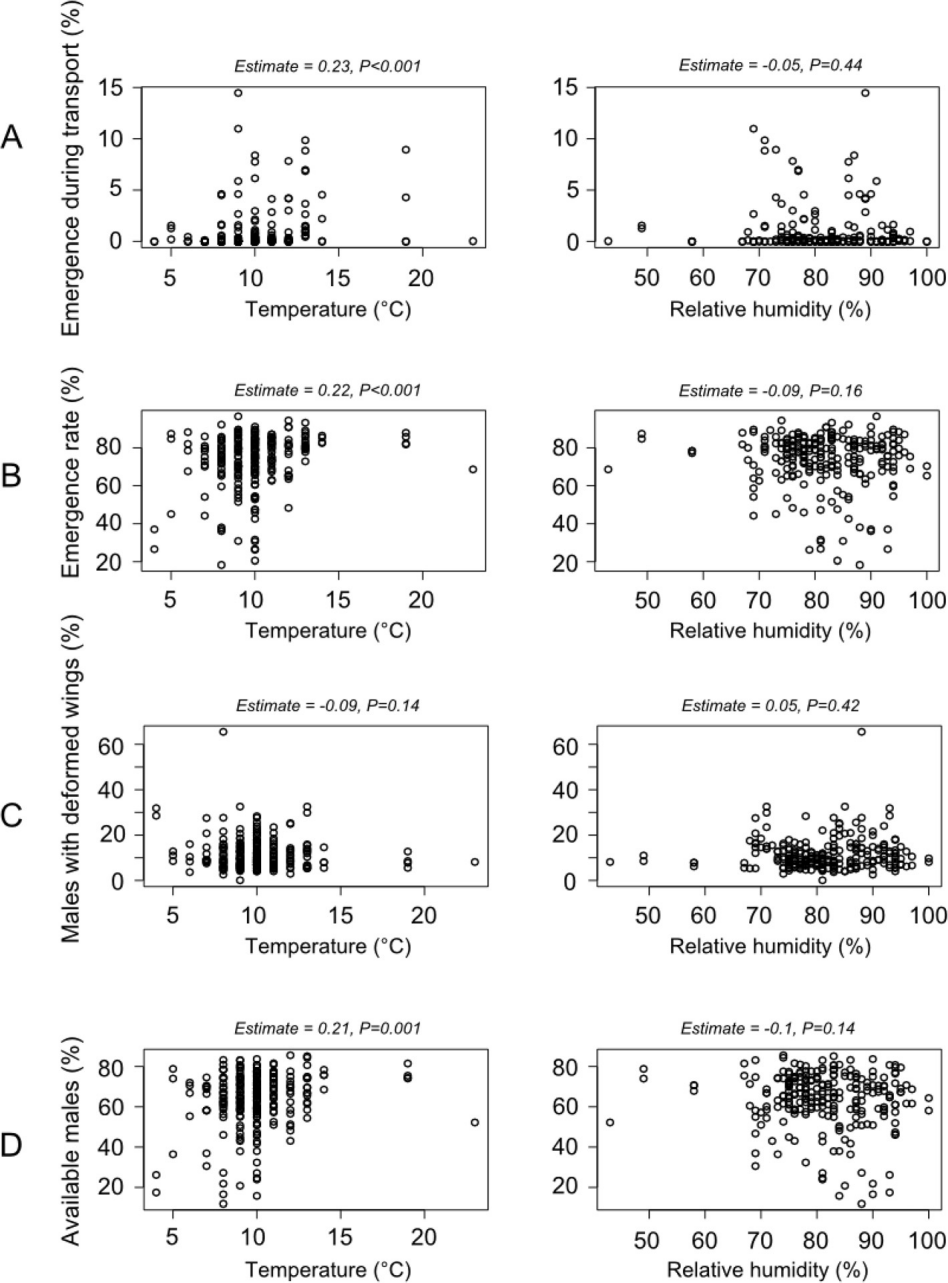
Correlation between the temperature and relative humidity during transport and entomological parameters. The values of the estimates and associated *P*-values correspond to the fix effects of the best mixed model for the corresponding response variable.

In 55% of shipments, fly emergence was observed during transport. Average fly emergence during transport was significantly higher for batch 1 pupae as compared with batch 2 pupae (*P* < 10^−3^; Table 1). The number of emerging flies increased with increasing temperatures and relative humidity inside the transport box (*P* < 10^−3^), showing that high temperatures stimulated emergence (Figure 3A). The interaction between temperature and relative humidity did not influence (*P* = 0.09) fly emergence during transport.

**Table 1:** **Effect of batch on flies emergences parameters (average ± sd)**

	**Comparison parameters**				
**Batch**	**Emergence during transport**	**Emergence in insectary**	**Emerged male with deformed wings**	**Mortality in insectary**	**Available sterile males**
**1**	0.9 ± 2.9^a^	72.2 ± 14.3^a^	12.0 ± 6.3^a^	5.9 ± 6.3^a^	61.7 ± 14.7^a^
**2**	0.7 ± 1.7^b^	76.1 ± 13.2^b^	10.7 ± 7.5^b^	5.8 ± 6.0^b^	65.8 ± 13.3^b^

*The data on the same column with a different letter are significantly different (P < 0.05).*

### Emergence of pupae at ISRA insectary

A total of 912,715 irradiated pupae were received in 132 shipments at the ISRA insectary (Dakar) from January 2011 to January 2014. Adult emergence was significantly higher for batch 2 pupae compared with pupae from batch 1 (*P* < 10^−3^; Table 1). Adult fly emergence at the ISRA insectary decreased with increasing temperatures and relative humidity during transport inside the insulated transport box (*P* < 10^−3^), showing that high temperatures were detrimental for adult emergence (Figure 3B). The interaction between temperature and relative humidity was not significant (*P* = 0.22). The frequency distribution of emerged flies by gender showed a small proportion (±sd) of females (3.3 ± 4.0%), which were systematically discarded because they have no useful role in the SIT for tsetse flies. The proportion of emerged male flies with deformed wings was statistically higher for batch 1 pupae compared with batch 2 pupae (*P* < 10^−3^; Table 1). The number of males emerging with deformed wings decreased with increasing temperatures and relative humidity inside the transport box (*P* < 10^−3^) but the correlation was not linear (Figure 3C). Their interaction had a positive effect (*P* < 10^−3^), showing that high temperatures and relative humidity were also detrimental.

### Survival of sterile males

After emergence, sterile male survival was monitored in the insectary for three to six days before being released in the field. The mortality rate of the sterile male flies was similar between batches (*P* = 0.43; Table 1). The climatic conditions during transport impacted survival of the male flies at the insectary: higher temperature and relative humidity during transport reduced survival after emergence (*P* < 10^−3^).

### Available sterile males for release

Taking into account the proportion of females, male flies with deformed wings, flies emerged during transport and the mortality rate in the insectary before release, the remaining sterile male flies (i.e. the male yield) were considered as available for release in the field for the eradication program. The average adult male yield was significantly higher for pupae from batch 2 than those from batch 1 (*P* < 10^−3^; Table 1). The temporal sterile male yield over the three years is shown in Figure 2. Any increase of temperature above the 10°C threshold during transport resulted in adult flies emergence during transport and higher mortality rates during the maintenance period at the insectary or a higher proportion of male flies with deformed wings. Temperatures below the 10°C threshold increased the proportion of male flies with deformed wings. Temperatures outside the 7-13°C range reduced the percentage of sterile males available for release. The impact of this variable was relatively linear up to 13°C (Figure 3D). The impact of humidity on the production of sterile males was not linear but bell-shaped in the range 75-95% (Figure 3D). All variation outside this range during transport was prejudicial to the flies. The significant interaction between temperature and relative humidity (*P* < 10^−3^) and the positive correlation between temperature and the percentage of available males showed that the negative effects of high temperatures on the proportion of operational males were offset by a higher relative humidity.

## Discussion

The tsetse eradication programme in the Niayes of Senegal adopted a novel approach for the SIT component i.e. the sterile flies used for the releases in the Niayes to eradicate the native *G. p. gambiensis* populations originated from a strain that was originally collected and mass-reared in a different country. The mature male pupae were irradiated and transported under chilling conditions to Senegal. To our knowledge this is the first time that such approach is implemented in tsetse eradication programme. In other SIT research projects, tsetse were transported as irradiated adults. Indeed, *G. tachinoides* adult males were reared at Maisons-Alfort and irradiated at Saclay (France), then transported by air to N’Djamena (Chad) to be release in low Logone (Cameroon) [21,22]. Whereas the transboundary shipment of mature irradiated pupae is applied for the first time in a tsetse eradication program with an SIT component, it is common practice for other pests such as fruit flies [23-25]. Under the umbrella of the PATTEC that calls for increased efforts of the African Heads of State and Government to better manage the tsetse fly and trypanosomosis problem, the Government of Senegal selected an AW-IPM eradication approach that included the SIT [26-28] as it is considered very efficient in eradicating riverine species [29]. To facilitate the implementation of the SIT component of the PATTEC initiative, two tsetse mass-rearing facilities were constructed, one located in West Africa (Burkina Faso) and the other one located in East Africa (Ethiopia), each having the projected capacity to produce one million sterile male tsetse per week (*G. p. gambiensis* in Burkina Faso and *G. pallidipes* in Ethiopia). Although the focus for both facilities is on their national tsetse programs, they might also be considered to produce sterile male flies for other potential programs that include an SIT component in the sub-region. This would require transport of pupae or adults (although these latter are more fragile for transport) of the target species from the mass-rearing facility to the requesting country. The long-distance transport of sterile male *G. p. gambiensis* pupae from Burkina Faso to Senegal over a period of three years in support of the eradication programme in the Niayes provided much insight on the effect of various parameters during the transport on the yield and quality of available sterile males for the SIT component of the campaign.

Based upon data from research carried out at the IPCL in Austria in support of the eradication campaign in the Niayes [30], a handling and transport protocol was proposed that took advantage of the differential development time of female and male pupae in combination with low temperatures. A temperature of ±10°C and RH of >75% was selected to transport the male pupae, in view that temperatures below 12°C prevent emergence of adult tsetse flies [31,32]. The chilled male pupae were transported in an insulated box that contained S8 phase change packs that maintained the temperature inside the box at around 10°C for the entire shipping period. Despite the delay in receiving some shipments usually due to technical reasons such as flight cancellation, the environmental conditions inside the parcel were maintained relatively stable for at least 72 hours.

The emergence rate of pupae received at the ISRA (average 74.2 ± 13.9%) was lower than what was observed at the source insectary in the CIRDES (between 90 to 95%, unpublished data). This difference might be related to vibrations or mechanical shocks during transport in addition to the stress due to the chilling, handling and irradiation of the pupae (tsetse pupae are specially sensitive the last days before adult emergence [32]), or to the effect of fluctuating temperature and/or humidity conditions during transport, or a combination of these factors. Although Vreysen *et al.* (1996) showed that treating 4–6 days old adult male *G. tachinoides*, *G. f. fuscipes* and *G. brevipalpis* decreased their average lifespan [33], Vreysen (1995) showed that irradiation of *G. tachinoides* pupae on day 25 or 28 post larviposition with doses up to 120 Gy did not negatively impact fly emergence [34]. Mutika *et al.* 2014 showed that emergence rate of *G. p. gambiensis* pupae that were chilled for 5 days and irradiated with 110 Gy during the first 24 h of cooling, had emergence rates between 76 to 91% [30]. Our data indicate that keeping the pupae at 10°C for two or three days did not negatively influence adult emergence rates. These results are in line with previous data showing that pupae of *G. p. gambiensis* stored at 10°C and 12.5°C for 3, 5 and 7 days [30], pupae of *G. morsitans* maintained at 12°C for 2 weeks [31], pupae of *G. pallidipes* stored at 15°C for 72 hours [13], pupae of *Haematobia irritans* Linnaeus kept at 4°C for up to 2 weeks [35] and pupae of *Cydia pomonella* kept at −0.16 to 0.61°C during transport from Canada to South Africa [36] had emergence rates that indicated no significant detrimental effect of the low temperatures.

The percentage of females flies was very low in the various consignments confirming the effectiveness of the method used at the source insectary to obtain only male pupae, namely by preventing emergence through cooling of the pupae when most of the female flies had emerged and the first males started emerging [37]. With this method it is possible to retain >97% of the female flies for colony production, which is essential for mass-rearing tsetse flies that have a very slow reproductive rate.

Between 10-12% of the male flies that emerged at the ISRA insectary had deformed wings. This anomaly was not observed at the CIRDES insectary (unpublished data). The same abnormalities were also reported with *G. tachinoides* males which were irradiated in France and airlifted to Chad for experimental releases [21]. It is postulated that these abnormalities could be correlated with variation of the temperature and relative humidity during transport and/or might also be related to vibrations or mechanical shocks during transport: this will require further research. During previous studies, a temperature below 16°C immobilized adults and did not allow the pre-imagoes to undergo normal development to reach the adult stage [32]. Accordingly, all the flies that emerged during transport had deformed wings in our study.

Mortality of sterile male flies at the ISRA insectary could be related either to handling stress or contamination of the blood as some flies died with their abdomen filled with blood. Deaths due to blood feeding could be caused by drugs administered to animals before slaughtering [38], or even by fluctuation of the blood temperature outside the optimal 35-37°C range during feeding [39]. The influence of temperature and relative humidity inside the insulated transport boxes on the survival of the emerged male flies in the insectary before release might be due to a depletion of fat reserves with higher temperatures.

Taking into account the number of the male flies emerged with deformed wings, that died in the ISRA insectary before release and the proportion of female flies in the consignments, the used handling and transport protocol gave a sterile male yield of 63.7 ± 14.1% as a proportion of the total pupae received. Performance of adults emerging from batch 1 pupae (chilled one day longer at 4°C) was significantly lower than those of batch 2 pupae, showing that the duration of chilling at 4°C had a negative impact. These available males (63.7%) were used to implement the operational release phase of the eradication project with very good results [40]. One of the key factors that determine the success of an AW-IPM program with an SIT component is the competitiveness of the released sterile males [41-43], and therefore, efforts are ongoing to further improve the transport conditions. The competitiveness of the released sterile male flies after release in the target zone is presently under evaluation in Senegal.

## Conclusions

This study confirms the feasibility of transporting chilled, irradiated male *G. p. gambiensis* pupae over long distances within a period of three days and also the available emerging adult male flies for the release phase of an eradication campaign with a SIT component. These results of the developed handling and transport protocol were very encouraging and have contributed to the success of the ongoing eradication phase in Senegal so far. The data collected will also be useful for future programs that will adopt the same approach.

## Additional file

Additional file 1:
**Mature sterile male pupae packaging protocol.**
