# Supplementary material for: Long distance transport of irradiated male Glossina palpalis gambiensis pupae and its impact on sterile male yield
Source: Parasit Vectors. 2015 May 1;8:259. doi: 10.1186/s13071-015-0869-3 (PMC4436170; doi:10.1186/s13071-015-0869-3)
Supplement: Additional file 1: — Mature sterile male pupae packaging protocol. [file 13071_2015_869_MOESM1_ESM.pdf]

Additional material

Mature sterile male pupae packaging protocol

The aim of this document is to present a standard packing and transport protocol of sterile male *Glossina palpalis gambiensis* pupae to stakeholders for tsetse area-wide integrated pest management programmes that include a sterile insect technique (SIT) component. Adherence to this protocol will prevent emergence of the male pupae inside the insulated box during transport, will reduce vibrations that have a negative impact on male fly quality and, will allow sufficient oxygenation of the pupae: strict adherence to the protocol will result in an optimal sterile male fly yield and in male flies with good sexual competitiveness.

First, to adequately oxygenate the pupae, and allow air to circulate between the petri dishes or cartons that contain the pupae, the amount of pupae should be adapted to the size of the petri dishes and the cartons.

We advise the following amounts of pupae:

- a maximum of 200 male *G. p. gambiensis* pupae for a petri dish with 5.4 cm diameter and 1.2 cm height (pierced with holes for air circulation)
- a maximum of 500 male *G. p. gambiensis pupae* for a petri dish with 8.8 cm diameter and 1.5 cm height (pierced with holes for air circulation)
- a maximum of 1500 male *G. p. gambiensis pupae* for a carton with a size of 12.4 x 8.2 x 2 cm (pierced with holes for air circulation).

Provisions should be made to reduce the vibrations and shocks received by the pupae during transport, i.e. a single layer of pupae should be placed on a layer of cotton and mosquito netting that are placed on the bottom of the petri dish or the carton, and covered by a second layer of cotton and mosquito netting (Figure 1). The carton or petri dish should be closed with adhesive tape taking care not to cover any aeration holes (Figure 1). The order of layers needs to be respected e.g. the second layer of cotton must be in direct contact with the pupae in order to prevent their accumulation on one side of the box should the transport box not be

placed upright during transport. Enough cotton should be placed in the petri dish or cartons so that a small pressure is exerted on the pupae that will stabilize them during transport.

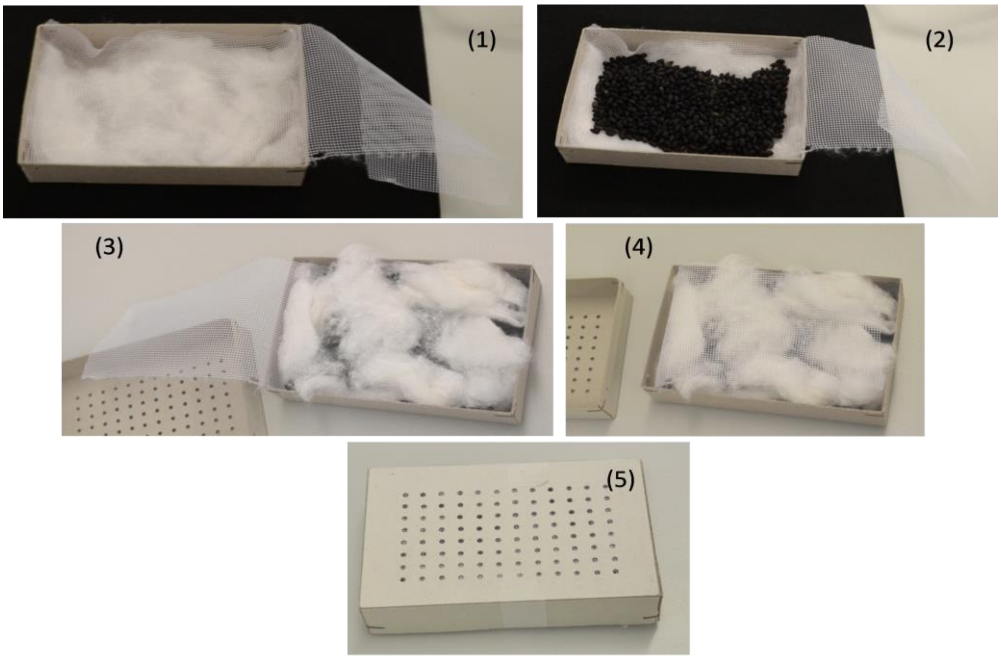

Figure 1: Packing of mature pupae in a carton (photos A. Parker, reproduced with permission).

Once the pupae are placed in these cartons, they are placed in an insulated transport box in the following way and order (Figure 2):

1) one S8 pack is placed on the bottom of the insulated box, and two are placed on the sides;

2) a bubble wrap or another shock absorber film is placed between the lateral S8 packs and the cartons or petri-dishes with the pupae, to reduce as much as possible vibrations; circular pieces of sponge could be also used but these must be glued to the cartons at least 48h before transport (to avoid potential solvent residues) or alternatively, using double sided adhesive tape (not shown on the figure);

3) pieces of polystyrene (or similar) are to be placed between the S8 packs and the walls of the transport container (top, bottom and sides) to fixate the packs so that they cannot vibrate against the cartons or Petri dishes;

4) a Hobo® data logger is placed inside the insulated transport box to record temperature and relative humidity during transport;

5) the top polystyrene piece is placed to cover the content of the container and the transport container is closed.

Four S8 packs can maintain the temperature inside the insulated box at 10 °C for up to 4 days.

Following the above mentioned guidelines, an insulated transport container of 25.5 x 21.5 x 26.5 cm can hold a maximum of 7500 *G. p. gambiensis* pupae. In case the duration of the transport is reduced to 2 days, one S8 pack can be replaced by 2 other pupal cartons, increasing the maximum number of pupae per insulate container to 10,500.

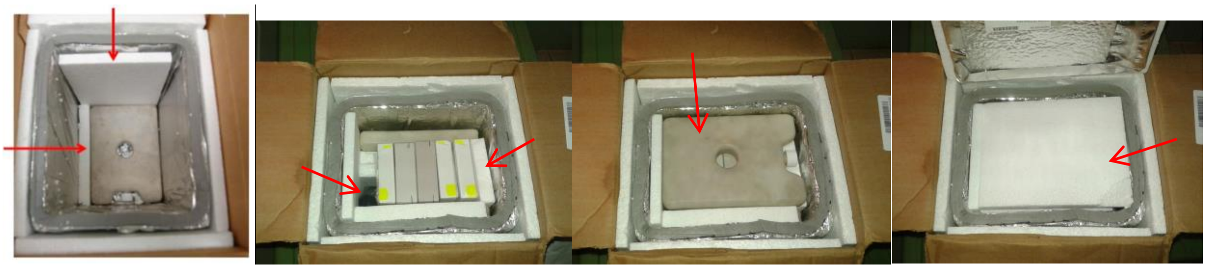

One S8 pack on the bottom of the transport container is fixated by 10 mm of EPS and a piece of 20 x 25 x 160 mm EPS at the side.

Two S8 packs placed on each long side of the transport box and held by 20 mm of EPS at each end of the transport box. Three or four cartons with pupae put inside with air space. A Hobo® data logger place inside the insulated box to record temperature and humidity.

Final S8 pack put on top of 20 mm pieces of EPS and fixated by 20 x 27 x 130 mm piece of EPS at the side.

A polystyrene sheet sits upon final S8 pack. Box lid sits on top.

Figure 2: Packing of the cartons holding the pupae in the transport container (photos G. Gimonneau). Cartons can also be placed horizontally.

Precautions have to be taken that the temperature does not increase during the irradiation of the pupae, as this will cause premature emergence. To maintain the temperature, the cartons containing the pupae can be placed in between two S8 packs during irradiation.

When pupae are irradiated in a facility other than the rearing facility, it is important not to break the cold chain to avoid premature emergence of the pupae. The transport box must be opened in a cold room at 5 °C, where the cylinder used in the irradiator (Figure 3) is stored, together with three S8 packs are placed around two cartons with the pupae.

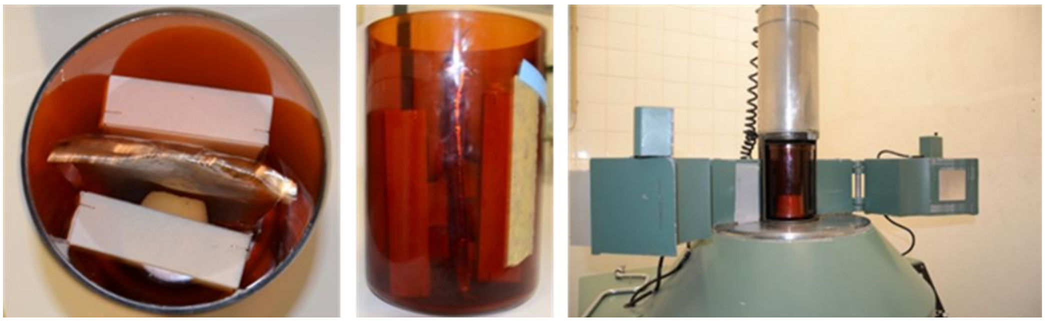

Figure 3: Irradiation conditions used to maintain the cold chain during irradiation.

Pupae can be transported either by air or by road. Transport by road is only advised if the transport takes less than 24 hours. Transport by air using courier services or air freight is recommended if the production and target areas are remote. Irrespective of the transport method chosen, total pupal transport time should not exceed 4 days.

Each consignment should be accompanied by proper documentation that should be attached to the outside of the transport box. Documents required are export permits, import permits, and certificates that certify that the content of the package does not pose a health hazard and has no commercial value. Other valuable data that should accompany each shipment: the number of pupae in the consignment, larviposition date, start of cooling of the pupae, radiation date and time, radiation dose, the shipping date and miscellaneous comments.
